# Legal responses to child endangerment on farms: Research methods

**DOI:** 10.3389/fpubh.2022.1015600

**Published:** 2022-11-10

**Authors:** Christopher P. Benny, Dorianne Beyer, Madeline Krolczyk, Barbara C. Lee

**Affiliations:** ^1^National Children's Center for Rural and Agricultural Health and Safety, Marshfield Clinic Research Institute, Marshfield, WI, United States; ^2^National and International Labor Standards Consultant, New York, NY, United States; ^3^Loyola University Chicago School of Law, Chicago, IL, United States

**Keywords:** child, endangerment, farm, agriculture, legal action, death, injury

## Abstract

In the US agriculture (including ranching) is among the most dangerous industries and it is the only one where children of any age are permitted in the worksite. Whether working or not, children are at risk of serious injury or death when present among the many hazards associated with agricultural work. In most cases the proximate cause of a traumatic incident involving a child (<18 years) is an adult's choice to allow the child's presence in a high-risk situation. Yet, little is known about the legal repercussions for a responsible adult when such events occur. With an overarching goal to enhance the culture of safety for children in agricultural settings, this project includes three phases: (1) identification and collection of public records and news reports regarding legal action following a childhood agricultural injury or fatality; (2) analysis of the proposed or imposed legal responses following these agricultural injuries and fatalities; and (3) development of recommendations for public agencies responding to events that lead to a criminal complaint or the imposition of non-criminal child welfare or other civil measures. This paper describes the project's mixed methods study design that yielded extensive details on 12 legal cases as well as perspectives from key informants on the strengths and limitations of legal responses to child endangerment on farms. Integration and analyses of data from quantitative and qualitative sources will be used to generate recommendations, including guidelines and protocols, for key stakeholder groups.

## Introduction

In the US, it is estimated that a child (<18 years) dies about every 3 days in a farm-related incident and that about 33 children are seriously injured every day on farms (1). Agriculture has been and continues to be one of the most dangerous occupational sectors according to the Bureau of Labor Statistics (2). The nationwide federal law does not set any federal age limitations for children who are working on family farms under the direction of parents or guardians (2). It is not only workers who are injured. In fact, about 60% of agricultural-related injuries are sustained by non-working children who are merely bystanders (1). A wide body of childhood agricultural injury literature is available from the US and many other countries. Updated statistics and several international commentaries are included in this special *Frontiers* issue.

Despite the frequency of childhood agricultural injuries and fatalities, responsible adults are rarely held accountable when a child is neglected or allowed in a dangerous setting. In launching this study, the team contacted numerous law schools, legal clinics and professional organizations to inquire on this topic, yet, virtually all respondents indicated they were not aware of legal cases involving child endangerment on farms, only of civil charges typically associated with equipment failure. To our knowledge, this is the first study of its kind. The goal of this project is to map legal cases where a parent/adult was prosecuted for child endangerment or neglect related to the death or serious injury of a child younger than 18 years. Specific aims include collecting data on actual legal cases; gathering perspectives of key informants and subject matter experts; interpreting findings from cases and informants; then generating recommendations for action. The reality is that family farm children are at an increased risk because they are on their home grounds, where they live and play contiguous with the worksite (3). Appropriate work assignments and keeping children separated from dangerous agricultural operations are required to mitigate risks. This project's end-goal is to influence the culture of safety in agricultural settings by raising awareness of adult accountability for safeguarding children from known dangers on a farm.

The term “accident” continues to be used by the general public but safety professionals prefer the term “incidents” as more reflective of the reality that all such events are preventable. The *2012 Blueprint for Protecting Children in Agriculture*—a national action plan, called for research that guided safety and health interventions for children in agriculture (4). While there have been many studies and interventions related to childhood safety on farms, there are no known publications or reports that assessed the legal consequences following serious farm-related injuries (which we defined as long lasting or permanent; the most common found was the loss of a full or partial limb) and fatalities on farms.

In broad outline, this project, which is a matter of first impression, collects, maps and analyzes legal cases where a parent/adult was subjected to legal consequences related to these childhood deaths or serious injuries in an agricultural setting, during the selected time period of 2015–2021. To gain further insights, interviews were conducted with key informants to solicit experiential perspectives on when and how legal consequences are determined and carried out. Conclusions and proposed recommendations will be reviewed with various stakeholders and advisors to gain their perspectives, raise awareness, and prompt action when indicated.

Knowing the sensitive nature of this study, the project team established several guiding principles to ensure fidelity to the overarching goal of better safeguarding children:

- a belief that all children deserve equal protection from harms, whether on a commercial or family farm or in agricultural or non-agricultural settings;
- the project's intended “audience” is public officials who are charged with responding to these fatalities or serious injuries; and
- exploring the greater use of civil penalties as alternatives to criminal prosecutions, such as community service, farm safety audits and parenting classes, have the potential to induce positive changes.

The study methods, protocols and data collection instruments were reviewed by the Institutional Review Board (IRB) of Marshfield Clinic Health Systems. The IRB approved the study as exempt because personal health information was not collected. This paper highlights the project's explanatory sequential mixed methods research design.

## Study design

**Figure 1:**
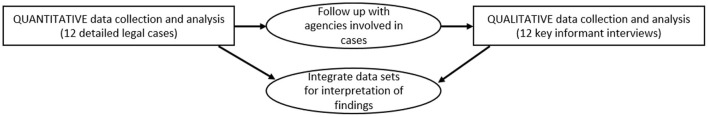
Mixed methods: Explanatory sequential design.

An explanatory sequential study design consists of first collecting and analyzing quantitative data then determining what type of qualitative data will help explain initial findings. Qualitative data is then collected and analyzed. The final step is integrating both data sets to interpret findings (5). As noted in Figure 1. This method provides a more holistic view of a situation, allowing both objective and subjective findings to form the basis of conclusions (6).

Quantitative data included demographic as well as legal data from publicly available sources. Child agricultural injury/fatality events were defined as non-fatal and fatal injuries that occurred on an agricultural work-site or involving agricultural machinery to those younger than 18 years. The “responsible parent/adult” was defined as the individual who was charged by law with being the onsite “supervisor” of the child victim. Those included parents, non-parental guardians or work supervisors at the time of the incident. Criteria for inclusion was a serious agriculture-related injury or fatality that resulted in some form of either proposed or imposed legal action against a responsible adult.

Qualitative data was gleaned from in-person meetings, telephone interviews, scheduled video meetings and e-communications with stakeholders, and advisors. The collected information was entered into a data collection tool (i.e., REDCap), which was used to fill pre-determined dataset fields to be analyzed by the project team. Integration of both data sets involved a series of telephone and in-person team meetings where discussions led to consensus on implications of individual cases and then generated conclusions based on the full dataset.

### Data sources

There is no comprehensive national database for either proposed or imposed legal action associated with childhood agricultural injuries and fatalities. Searches for cases were primarily conducted using federal and state Departments of Labor, federal and state Occupational Safety and Health Administrations, law offices, law schools, district attorney associations, child welfare agencies, offices of state Attorneys General, offices of local district attorneys, Lexis+, Westlaw, AgInjuryNews.org (7), Google Alerts, and internet searches.

#### Lexis+

Lexis+ is a subsection of LexisNexis, a legal research database for legal case law research. The project team used the advanced search option with keywords to search for cases. The keywords used were, “Prosecution, Child, Agriculture, Injury, Fatality, Child Labor, Negligence, Legal Action, and Child Protective Services.” When potential cases were identified, the tabs citing decisions or other citing sources allowed the project team to assess if the incident met inclusion criteria.

#### Westlaw edge

Westlaw edge is a database for case law research from Westlaw. The project team used the advanced search option with keywords to search for cases. The same keywords were used as in Lexis+ and then applied to searches among secondary sources including trial court orders, statutes and court rules, regulations, briefs, and proposed and enacted legislation.

If sufficient data could not be identified from Lexis+ or Westlaw Edge additional internet searches, news media article searches, and court record database searches were conducted to assess if the incident met study inclusion criteria. Of the sources used, cases that met the selection criteria were only found using AgInjuryNews.org, Google Alerts and internet searches.

#### AgInjuryNews.org

The AgInjuryNews.org website is a publicly accessible free database of agricultural injuries and fatalities. Using the date, country/region, and age filters on the website the reports were filtered to display only relevant cases. The project team visited the source uniform resource locator (url) for the filtered cases to determine whether the case met study criteria. Google searches were conducted for the identified cases to look for additional data on each case.

#### Google alerts

Google Alerts is a publicly accessible free service offered by Alphabet Inc. to monitor the internet for new content. The project team created a google alert using keywords such as- “Prosecution, Child, Agriculture, Injury, Fatality, Child Labor, Negligence, Legal Action, and Child Protective Services.” The alert was set to provide results once a day and a filter was applied to limit the results to the US. When a childhood agricultural injury or fatality event was identified by the alert, the project team reviewed the source article and conducted Google searches to seek additional data.

#### Internet searches

The project team conducted internet searches using browsers such as “Google,” “Internet Explorer,” “Microsoft Edge,” and “Yahoo” to locate data on childhood agricultural injury and fatality events. Once cases were identified, internet searches were used to find publicly available court records and additional news articles for our data collection process. Additionally, first person perspectives on criminal charges and their consequences were secured from department of labor representatives, district attorneys, Mothers Against Drunk Driving (8) representatives, and news reporters.

## Data

### Selection criteria

**Figure 2:**
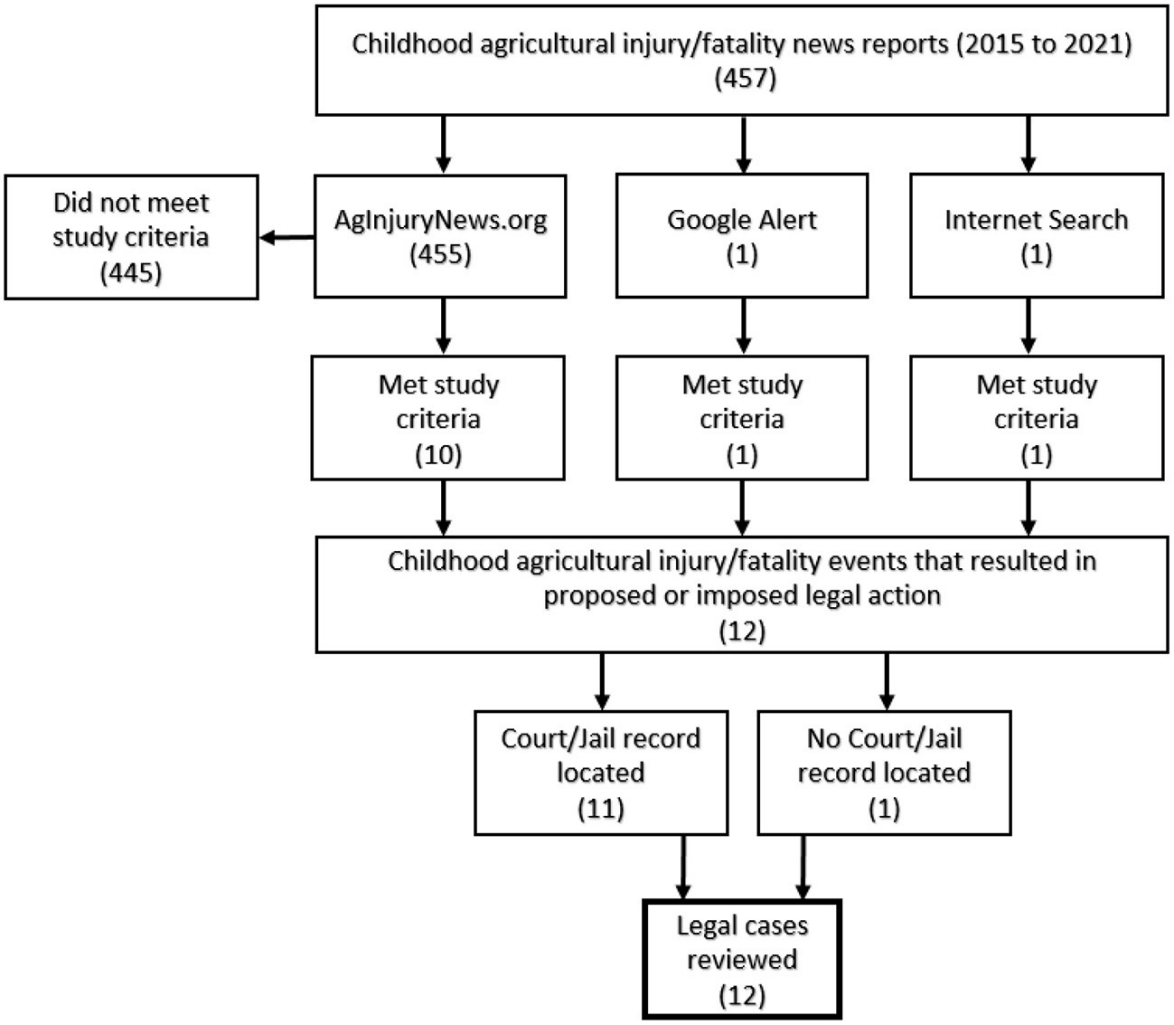
Legal case identification.

Due to the retrospective nature of the study, this project did not recruit participants. All data collected on the subjects (the responsible parent/adult) were available through public sources. If the data identification source met selection criteria (only US cases from 2015–2021 involving youth younger than 18 years on farms and ranches), an in-depth search was conducted using news articles and court records to obtain data. The minimum dataset consisted of date, location of incident, injury agent, age of victim, name of person responsible and their relationship to the victim, and the proposed or imposed legal action. A case would be excluded if, for example, a public record or news article mentioned an ongoing investigation but no subsequent information was located regarding an ensuing criminal or civil complaint. See Figure 2. Cases meeting all criteria were entered into the custom-designed REDCap data collection tool. Cases with incomplete data or from international sources were filed separately for potential, later reference.

### Court records

Court records in the US are generally publicly available (9). However, sealed court documents, closed hearings and sensitive personal information are more the rule when involving children or medical information. This hampered many search outreaches.

Publicly available court records were located using state-specific online portals. The project team searched for publicly available court records corresponding to the identified cases by identifying the online portal for the state where the incident occurred. Most portals required information such as “first name,” “last name,” “county,” and a date range for when the case was filed. Using these data points the publicly available court records for the identified cases were located.

### REDCap data collection tool

REDCap is a secure software used to create and manage surveys and databases (10). A REDCap data collection tool was created by the project team and consisted of 57 fields. Each identified child agricultural injury/fatality event was assigned a project specific case number. The case number was a four-digit numeric field. The first two digits corresponded to the sequence in which the event was found and the last two digits corresponded to the year that the event occurred. For example- the first event found in 2015 would be labeled “0115,” the next event would be labeled “0215.” Other fields were narrative text fields, portable document format (pdf) uploads, date field, button lists (select one of the following), and checkbox lists (selecting from multiple options). The data collection tool consisted of 35 text fields, 12 file upload fields, seven button lists, two checkbox lists, and one date field. The use of the REDCap data collection tool ensured that the data abstraction process for the identified cases was conducted in a consistent fashion, free from bias. On average, the project team spent about 1 h analyzing all court documents and news reports for each case to understand the legal terminology and ensure consistency in REDCap data abstraction. A final quality review included a 2-day in-person meeting where the REDCap data set for each case was reviewed, allowing for a final check for errors. Common as well as unique findings across the cases were documented separately and then used to generate questions for key informants.

### Qualitative data collection

For qualitative data the collection process included methods to secure information, resources, and perspectives from individuals in positions to provide insights on specific cases and/or related issues such as actions taken by child welfare agencies, working in collaboration with legal entities. Methods included presentations, in-person meetings, telephone interviews, video meetings, and e-communication. The project team took notes from each event then subsequently discussed their relevance for achieving study objectives.

#### Phone contacts

The project's legal consultant created a cover sheet for phone calls to potential data sources and trained the research coordinator on how to conduct phone interviews with lawyers and public officials. The research coordinator compiled lists of law offices that focused on agricultural injuries, non-profit organizations that focused on child safety such as Mothers Against Drunk Driving (MADD), and law schools with courses or clinics that specialized in agricultural law. The research coordinator and the legal consultant contacted these potential informants to request information on these types of injury/fatality events. In addition, individuals with experience in the field were invited to join a panel of experts subsequent to data collection to contribute to proposed recommendations for action.

#### E-communications

The project team compiled a list of the Directors of the states' child welfare agencies (also known as Child Protective Services) and a list of the District Attorneys Associations or equivalent. Regarding child welfare agencies, an initial email was sent to 62 individuals who held leadership roles in all 50 States and two US territories. After 2 weeks, only three responses (1.5%) were received, thus, a reminder email with “read receipt” was sent. The read receipts indicated that of the 59 reminder emails 21 (36%) individuals had read the email but did not provide a response. Four (7%) of DA associations responded to the email, of whom three provided relevant information for the project. Two agencies agreed to video meetings for further discussion.

Regarding District Attorney Associations, email addresses were secured for 44 states' district attorney associations or equivalent. An initial request for information was sent to 42 state-specific district attorney associations (or their equivalent) in the US; then after 2 weeks a reminder email was sent with a read receipt to the unresponsive associations. Only 19 (45%) associations read the email and, of these, four responded with a reply. This e-communication method yielded no data on additional cases, but some did offer valuable resources applicable to the project's recommendations. Electronic mail was also used to follow up with journalists and district attorneys for the cases that were already identified to either secure missing information or to look for additional cases.

#### Key informant interviews

There was no standardized questionnaire or interview tool relevant to this study, thus, the team pilot tested a series of questions. After each use, the set of questions was condensed and refined. The interviews aimed to seek a better understanding of the legal actions that were being taken on these incidents as well as the roles of various agencies. Additionally, the interviews yielded perspectives regarding the rarity of occurrences that met project criteria—in contrast to other types of child harm, endangerment or neglect.

A total of 12 Zoom online video meetings (11) were conducted with district attorneys, law professors, department of labor representatives, child welfare personnel, and child death review experts. Interview questions were emailed to interviewees at least 1 week prior to the meeting. Table 1 provides the primary interview questions. Each session began with a brief project overview with slides, as a means to share the study's purpose and guiding principles as well as to minimize bias in responses.

**Table 1:** Interview questions submitted to key informants.

1.	Have you/your office had any experiences responding to a report that a child was killed or seriously injured on a farm? If so, can you briefly describe your experience?
2.	Have you/your office been involved in a legal response (action/decision) following a serious injury or death of child associated with an agricultural worksite or farm machinery? If yes, please explain (e.g., culpability, charges, penalties, contributing circumstances, etc.)
3.	With about 100 preventable child farm deaths each year, typically with an adult present, why do you think so few resulted in any criminal charges or civil complaints? How does this compare to non-farm cases of child deaths?
4.	The next project phase includes drafting recommendations for response by public agencies. Would you/your office be willing to review and comment on these?
5.	What else should we know about public agencies and their response to negligent adult practices that result in a child's death or serious injury in an agricultural setting?

## Analysis

Despite extensive searches and inquiries, only 12 legal (criminal) cases were identified that met study criteria from 2015 to 2021 in the U.S. After cases were documented into REDCap the project team thoroughly reviewed each case to identify variables of those charged and those not charged with a criminal offense or subjected to a civil disposition. Additionally, the project team identified which types of action (i.e., criminal, civil, or child welfare) were most commonly involved. During this process, the team entered notes requiring follow-up (e.g., pending court date) into the comments section of the REDCap tool. From these analyses, preliminary findings were collated across all cases and timelines were set to follow-up on pending actions.

News reports and court records provided information on roles of various stakeholder groups, including public enforcement agencies, incident scene investigators, private legal offices, child welfare agencies, advocacy groups and others. From this list, the potential participants for key informant interviews was generated. The scheduled video meetings provided a range of insights on legal actions taken (or not taken). Equally important, interviews with child welfare agency personnel yielded valuable perspectives about approaches used in “hypothetical” cases, given that their data on child neglect and maltreatment is confidential.

This project's final phase will use findings and input from stakeholders and advisors to draft recommendations for action by first responders to injury events, district attorneys, child welfare staff, reporters and advocacy groups. Invitational roundtable discussions with stakeholders will consider next steps such as development of guidebooks to upgrade and amplify the protocols and practices of those public agencies when responding to these injury/fatality events, along with discipline-specific training *via* online sessions and in-person workshops. A final report that presents the study's overall findings and anticipated impact will be prepared and disseminated among relevant stakeholder groups, including, amongst others, the agricultural media and public and private law organizations.

## Limitations

Several limitations were encountered and should be noted. During the data collection process, many news reports of incidents were located, indicating an investigation was pending; but there were no subsequent legal charges were filed. It was not possible to determine if further action was taken outside of the legal system such as child welfare services, or if the case investigation was simply closed. The cases that met the project criteria represent a very small subset of childhood agricultural injuries and fatalities. Another limitation was the lack of published literature on this topic, as well as the minimal guidance available from schools of law or legal practices. Thus, findings from this project cannot be compared to similar studies in the US or elsewhere.

## Discussion and conclusion

This novel project addressed an issue that has the potential to influence the culture of safety affecting children living, working and visiting on farms and ranches. New methods were tested and adopted to gather data and gain perspectives on the legal response to child endangerment on farms. The difficulty in locating cases that met study criteria suggests that legal actions imposed on culpable adults are relatively rare in contrast to the significant number of preventable childhood agricultural injuries and deaths reported in news articles.

The project team does not seek to establish or inspire further punishment or community opprobrium of those responsible adults. Rather, much like the principles of restorative justice (https://restorativejustice.org/), penalties such as community service or training in farm safety aim to provide a measure of healing for the responsible party. Findings will be used to identify opportunities for improving the responses of the relevant governmental enforcement agencies following the injury or death of a child on a farm. The end goal is to raise awareness in agricultural communities about the civil and criminal consequences of child endangerment in work settings as a means to enhance the culture of safety for all children in agricultural settings.

## Data availability statement

Publicly available datasets were analyzed in this study. This data can be found here: https://www.aginjurynews.org/.

## Ethics statement

The studies involving human participants were reviewed and approved by Marshfield Clinic Research Institute Institutional Review Board (IRB). Written informed consent for participation was not required for this study in accordance with the national legislation and the institutional requirements.

## Author contributions

CB and BL prepared the initial draft and subsequent revisions of this manuscript. DB and MK contributed equally to the review and refinement of the manuscript's description of methodology for conducting this study. All authors contributed to the article and approved the submitted version.

## Funding

Funding for this project was provided by the Dean Emanuel Endowment fund to the National Farm Medicine Center, Marshfield, WI, a non-for-profit program of the Marshfield Clinic Health System.

## Conflict of interest

The authors declare that the research was conducted in the absence of any commercial or financial relationships that could be construed as a potential conflict of interest.

## Publisher's note

All claims expressed in this article are solely those of the authors and do not necessarily represent those of their affiliated organizations, or those of the publisher, the editors and the reviewers. Any product that may be evaluated in this article, or claim that may be made by its manufacturer, is not guaranteed or endorsed by the publisher.
